# Supplementary material for: Spectroscopic-network-assisted precision spectroscopy and its application to water
Source: Nat Commun. 2020 Apr 6;11:1708. doi: 10.1038/s41467-020-15430-6 (PMC7136255; doi:10.1038/s41467-020-15430-6)
Supplement: Supplementary file 1 — Supplementary Information [file 41467_2020_15430_MOESM1_ESM.pdf]

# Supplementary Information

# Spectroscopic-network-assisted precision spectroscopy and its application to water

Tóbiás et al.

**Supplementary Note 1 : Network-theoretical glossary**

Measured (or computed) rovibrational transitions can be treated as elements of spectroscopic networks. Spectroscopic networks[1–3] are weighted, directed, loop-free multigraphs, where (a) the vertices (nodes) are energy levels, (b) the edges (links) are rovibronic transitions, oriented from their lower energy levels to their upper ones, and (c) nonnegative edge weights are assigned to the transitions (these weights are chosen for the actual task the spectroscopic network is applied for). To process all the information the rovibronic lines contain, it is necessary to analyze the spectroscopic networks for (a) components (collections of energy levels not connected by transitions), (b) paths (sequences of connected, unrepeated lines and states), (c) cycles (series of connected transitions and energy levels, where every rovibronic state has two neighboring energy levels), and (d) bridges (lines whose deletion increases the number of components) without relying on the edge directions.

A path gives a prediction, with a well-defined uncertainty, for the energy difference between its starting and ending points:

$$E_{M_{L+1}} - E_{M_1} = \sum_{k=1}^{L} S_k \sigma_{I_k}, \tag{1}$$

$$u(E_{M_{L+1}} - E_{M_1}) = \sqrt{\sum_{k=1}^{L} u^2(\sigma_{I_k})}, \tag{2}$$

where (a) $L$ is the length of the path, (b) $I_1, I_2, ..., I_L$ and $M_1, M_2, ..., M_{L+1}$ are the indices of transitions and energy levels participating in this path, respectively, (c) $E_i$ is the energy value of the $i$th rovibronic state, and (d) $\sigma_{I_k}$ is the wavenumber of the $I_k$th line, (e) $u(\sigma_{I_k})$ is the uncertainty of $\sigma_{I_k}$, and (e) $S_{I_k}$ is the sign assigned to the $I_k$th transition in this path. If the starting point is the lowest-energy state of the molecule, then this predicted energy difference corresponds to a predicted rovibronic energy value of the ending point.

Cycles are extremely useful when compatibility of the transitions and their uncertainties are investigated. If the path described in Supplementary Equations 1–2 is completed to a cycle with an extra line of index $I_{L+1}$, we have two estimates for $E_{M_{L+1}} - E_{M_1}$. The absolute difference of these estimates is the discrepancy of the investigated cycle, formulated as[4]

$$D = \left| \sum_{k=1}^{L+1} S_k \sigma_{I_k} \right| \tag{3}$$

and augmented with a definitive uncertainty,

$$u(D) = \sqrt{\sum_{k=1}^{L+1} u^2(\sigma_{I_k})}. \tag{4}$$

For the discrepancies, a Student-$t$ test can be performed, prescribing

$$D \leq t_{\text{crit}} u(D), \tag{5}$$

where $t_{\text{crit}}$ is the critical Student-t factor ($t_{\text{crit}} \approx 2$ for 95 % significance level). If a cycle does not satisfy Supplementary Equation 5, it means that a conflict occurs among the associated lines. Note that repeated measurements can be viewed as cycles of length two.

Since the energy levels of the various components are not connected by definition, we need to relate the energy values of the states to the energies of the lowest-lying energy levels (cores) of their components. When the core of a component is the lowest-energy level of a nuclear-spin isomer of the molecule, this component is a principal component, otherwise it is a floating component.

Due to the fact that the vertex degrees (edge counts of the individual nodes) of a spectroscopic network follow an inverse-power-like (heavy-tailed or quasi scale-free[5]) distribution[1], they exhibit only a small number of hubs (high-degree energy levels). Thus, the accurate determination of the energy values of hubs is of utmost importance for an improved characterization of spectroscopic networks.

As energy levels beyond a bridge cannot be known more accurately than the bridge itself, a bridge may compromise the accuracy of the derived energy levels. Therefore, experimentalists should pay special attention to determine these bridges as precisely as possible.

For each component, there is a minimal subset of transitions (spanning tree), making all the energy levels of the component connected and helping to reduce the number of new measurements. The set of the selected spanning trees corresponds to a spanning forest of the spectroscopic network. All the lines outside a spanning forest define basic cycles, whose collection (including all the non-bridge transitions) is a cycle basis. Since all the cycles can be expressed with the combination (symmetric difference) of basic cycles, construction of such cycle bases and evaluation of their entries could be sufficient to test the compatibility of rovibronic lines [see Supplementary Equation 5].

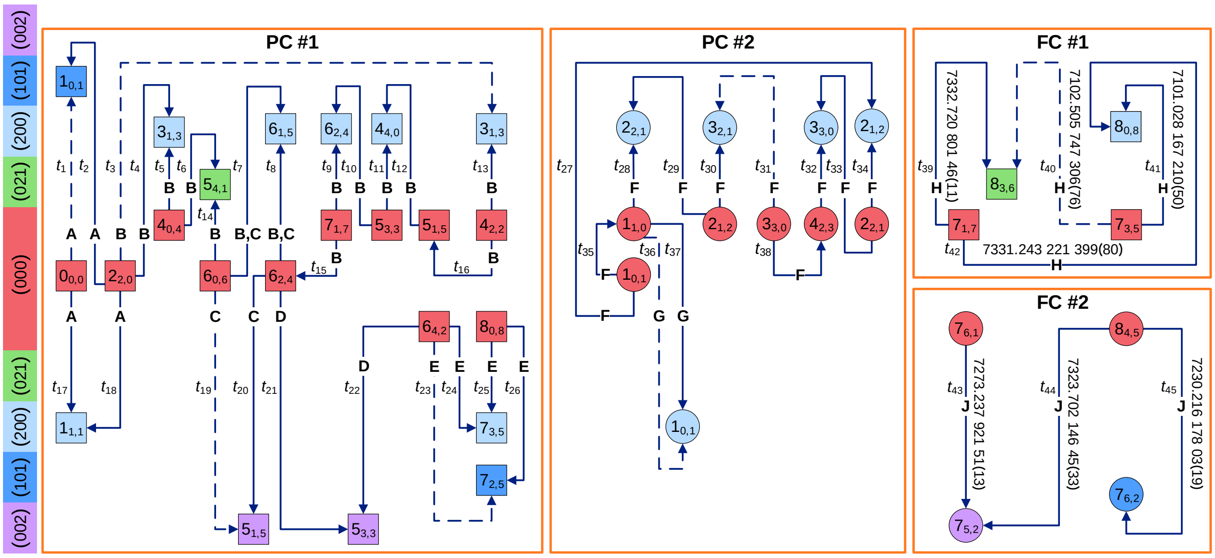


**Supplementary Figure 1**: **Graphical illustration of the most important network-theoretical notions, for the case of $H_2{}^{16}O$, based on a sample network of some lines ($t_1$,$t_2$,...,$t_{45}$) selected from files sd1.txt and sd2.txt.** The rovibrational states are labelled with $(v_1v_2v_3)J_{K_a,K_c}$, where $(v_1v_2v_3)$ contains the normal-mode quantum numbers (following the Mulliken convention[6]) and $J_{K_a,K_c}$ corresponds to the asymmetric-top quantum numbers[7]. The energy levels are placed palindromically in increasing (upper half) and descreasing (lower half) energy order of their vibrational parents and distinguished with different colors for improved transparency. The $J_{K_a,K_c}$ rotational labels are indicated individually for each rovibrational state, while the $(v_1v_2v_3)$ vibrational assignments are listed in the left-side color legend. PC #1 and PC #2 are principal components of the sample network, where the lowest-energy states of the *ortho* and *para* nuclear-spin isomers, $(000)1_{0,1}$ and $(000)0_{0,0}$, respectively, are included. The other components (FC #1 and FC #2) are floating components. Transitions with label J form a path, while those with labels A, B, C, E, F, G, and H represent seven cycles. Lines with label D are bridges, as removing any of them breaks PC #1 into two components. Taking the line positions and the related uncertainties of $t_{43}$, $t_{44}$, and $t_{45}$ from files sd1.txt and sd2.txt , the energy difference of the starting $[(000)7_{6,1}]$ and ending $[(101)7_{6,2}]$ points together with its uncertainty can be given as follows [see Supplementary Equations 1–2]:

$$7273.23792151 - 7323.70214645 + 7230.21617803 = 7179.75195309 \text{ cm}^{-1},$$

$$\sqrt{13^2 + 33^2 + 19^2} \times 10^{-8} = 4.0 \times 10^{-7} \text{ cm}^{-1}.$$

Similarly, utilizing the wavenumbers of transitions $t_{39}$, $t_{40}$, $t_{41}$, and $t_{42}$, the discrepancy of cycle H and its uncertainty can be obtained in the following way [see Supplementary Equations 3–4]:

$$7332.72080146 - 7102.505747306 + 7101.028167210 - 7331.243221399 = 3.5 \times 10^{-8} \text{ cm}^{-1},$$

$$\sqrt{110^2 + 76^2 + 50^2 + 80^2} \times 10^{-9} = 1.64 \times 10^{-7} \text{ cm}^{-1}.$$

It can be recognized that this discrepancy and its uncertainty obeys Supplementary Equation 5. In cycle G of length two, $t_{37}$ is a repeated measurement of $t_{36}$. Based on the relative energies in file sd4.txt, the lowest-energy states (cores) of the four components are $(000)0_{0,0}$, $(000)1_{0,1}$, $(000)7_{1,7}$, and $(000)8_{4,5}$. The energy differences among the cores cannot be calculated from lines of the sample subnetwork: further transitions are needed to link FC #1 and FC #2 with PC #1 and PC #2, respectively.

**Supplementary Figure 1**: **continued from previous page**

The vertex degree of $(000)2_{2,0}$ is 4 as it is incident to four rovibrational lines. All the $(000)$ rotational levels of this sample network are high-degree nodes (hubs) of the spectroscopic network built upon the IUPAC database[8] of all the available experimental rovibrational lines of $H_2{}^{16}O$. The continuous arrows specify a spanning tree for each (PC #1, PC #2, FC #1, and FC #2) component. These spanning trees contain lines visiting all the energy levels and provide a spanning forest for the sample network. The lower and upper states of a dashed line (such as $t_1$) are connected by a unique path within the spanning forest (in this example, $t_2 - t_{18} - t_{17}$), resulting in a basic cycle (in this case, cycle A) associated to this spanning forest. The cycle basis[4] of this spanning forest consists of seven basic cycles (A, B, C, E, F, G, and H) determined by the dashed lines. The symmetric difference of basic cycles B and C is the union of B and C without their common lines, $t_7$ and $t_8$. This symmetric difference ($t_4$–$t_5$–$t_6$–$t_{14}$–$t_{19}$–$t_{20}$–$t_{15}$–$t_9$–$t_{10}$–$t_{11}$–$t_{12}$– $t_{16}$–$t_{13}$–$t_3$) corresponds to another cycle of the sample network.

There are possibilities to restrict the spanning forest in such a way that various conditions hold for the edge weights. A feasible restriction is that each path of the spanning forest, leading to a core, should be the shortest possible (i.e., the sum of the edge weights is minimal in this path). This shortest-path-based spanning forest can be obtained with Dijkstra's procedure[9].

The network-theoretical terms introduced in this section can be understood on a sample network depicted in Supplementary Figure 1. In the caption to this figure, worked examples are presented for the application of Supplementary Equations 1–4, as well.

## Supplementary Note 2 : Effective Hamiltonian modeling

The $(000)$ rotational energy levels of $H_2{}^{16}O$ can be characterized, for low $J$ values[10], with the following asymmetric-top Watson-type effective rotational Hamiltonian operator (employing A-reduction and the $\mathrm{I^r}$ representation)[11,12]:

$$\hat{\mathbf{H}} = \hat{\mathbf{H}}_2 + \hat{\mathbf{H}}_4 + \hat{\mathbf{H}}_6 + \hat{\mathbf{H}}_8 + \hat{\mathbf{H}}_{10} + \hat{\mathbf{H}}_{12} + \hat{\mathbf{H}}_{14} + ..., \tag{6}$$

where

$$\hat{\mathbf{H}}_2 = A\hat{\mathbf{J}}_z^2 + B\hat{\mathbf{J}}_x^2 + C\hat{\mathbf{J}}_y^2, \tag{Ta}$$

$$\hat{\mathbf{H}}_4 = -\mathit{\Delta}_{\mathrm{K}}\hat{\mathbf{J}}_z^4 - \mathit{\Delta}_{\mathrm{JK}}\hat{\mathbf{J}}^2\hat{\mathbf{J}}_z^2 - \mathit{\Delta}_{\mathrm{J}}\hat{\mathbf{J}}^4 - \{\delta_{\mathrm{K}}\hat{\mathbf{J}}_z^2 + \delta_{\mathrm{J}}\hat{\mathbf{J}}^2, \hat{\mathbf{J}}_{xy}^2\}, \tag{Tb}$$

$$\hat{\mathbf{H}}_6 = H_{\mathrm{K}}\hat{\mathbf{J}}_z^6 + H_{\mathrm{KJ}}\hat{\mathbf{J}}^2\hat{\mathbf{J}}_z^4 + H_{\mathrm{JK}}\hat{\mathbf{J}}^4\hat{\mathbf{J}}_z^2 + H_{\mathrm{J}}\hat{\mathbf{J}}^6 + \{h_{\mathrm{K}}\hat{\mathbf{J}}_z^4 + h_{\mathrm{JK}}\hat{\mathbf{J}}^2\hat{\mathbf{J}}_z^2 + h_{\mathrm{J}}\hat{\mathbf{J}}^4, \hat{\mathbf{J}}_{xy}^2\}, \tag{Tc}$$

$$\hat{\mathbf{H}}_8 = L_{\mathrm{K}}\hat{\mathbf{J}}_z^8 + L_{\mathrm{KKJ}}\hat{\mathbf{J}}^2\hat{\mathbf{J}}_z^6 + L_{\mathrm{JK}}\hat{\mathbf{J}}^4\hat{\mathbf{J}}_z^4 + L_{\mathrm{JJK}}\hat{\mathbf{J}}^6\hat{\mathbf{J}}_z^2 + L_{\mathrm{J}}\hat{\mathbf{J}}^8 \\ + \{l_{\mathrm{K}}\hat{\mathbf{J}}_z^6 + l_{\mathrm{KJ}}\hat{\mathbf{J}}^2\hat{\mathbf{J}}_z^4 + l_{\mathrm{JK}}\hat{\mathbf{J}}^4\hat{\mathbf{J}}_z^2 + l_{\mathrm{J}}\hat{\mathbf{J}}^6, \hat{\mathbf{J}}_{xy}^2\}, \tag{Td}$$

$$\hat{\mathbf{H}}_{10} = P_{\mathrm{K}}\hat{\mathbf{J}}_z^{10} + P_{\mathrm{KKJ}}\hat{\mathbf{J}}^2\hat{\mathbf{J}}_z^8 + P_{\mathrm{KJ}}\hat{\mathbf{J}}^4\hat{\mathbf{J}}_z^6 + P_{\mathrm{JK}}\hat{\mathbf{J}}^6\hat{\mathbf{J}}_z^4 + P_{\mathrm{JJK}}\hat{\mathbf{J}}^8\hat{\mathbf{J}}_z^2 + P_{\mathrm{J}}\hat{\mathbf{J}}^{10} \\ + \{p_{\mathrm{K}}\hat{\mathbf{J}}_z^8 + p_{\mathrm{KKJ}}\hat{\mathbf{J}}^2\hat{\mathbf{J}}_z^6 + p_{\mathrm{JK}}\hat{\mathbf{J}}^4\hat{\mathbf{J}}_z^4 + p_{\mathrm{JJK}}\hat{\mathbf{J}}^6\hat{\mathbf{J}}_z^2 + p_{\mathrm{J}}\hat{\mathbf{J}}^8, \hat{\mathbf{J}}_{xy}^2\}, \tag{Te}$$

$$\hat{\mathbf{H}}_{12} = Q_\mathrm{K}\hat{\mathbf{J}}_z^{12} + Q_\mathrm{KKKJ}\hat{\mathbf{J}}^2\hat{\mathbf{J}}_z^{10} + Q_\mathrm{KKJ}\hat{\mathbf{J}}^4\hat{\mathbf{J}}_z^8 + Q_\mathrm{JK}\hat{\mathbf{J}}^6\hat{\mathbf{J}}_z^6 + Q_\mathrm{JJK}\hat{\mathbf{J}}^8\hat{\mathbf{J}}_z^4 + Q_\mathrm{JJJK}\hat{\mathbf{J}}^{10}\hat{\mathbf{J}}_z^2 \tag{Tf}$$
$$+ Q_\mathrm{J}\hat{\mathbf{J}}^{12} + \{q_\mathrm{K}\hat{\mathbf{J}}_z^{10} + q_\mathrm{KKJ}\hat{\mathbf{J}}^2\hat{\mathbf{J}}_z^8 + q_\mathrm{KJ}\hat{\mathbf{J}}^4\hat{\mathbf{J}}_z^6 + q_\mathrm{JK}\hat{\mathbf{J}}^6\hat{\mathbf{J}}_z^4 + q_\mathrm{JJK}\hat{\mathbf{J}}^8\hat{\mathbf{J}}_z^2 + q_\mathrm{J}\hat{\mathbf{J}}^{10}, \hat{\mathbf{J}}_{xy}^2\},$$
$$\hat{\mathbf{H}}_{14} = R_\mathrm{K}\hat{\mathbf{J}}_z^{14} + R_\mathrm{KKKJ}\hat{\mathbf{J}}^2\hat{\mathbf{J}}_z^{12} + R_\mathrm{KKJ}\hat{\mathbf{J}}^4\hat{\mathbf{J}}_z^{10} + R_\mathrm{KJ}\hat{\mathbf{J}}^6\hat{\mathbf{J}}_z^8 + R_\mathrm{JK}\hat{\mathbf{J}}^8\hat{\mathbf{J}}_z^6 + R_\mathrm{JJK}\hat{\mathbf{J}}^{10}\hat{\mathbf{J}}_z^4 \tag{Tg}$$
$$+ R_\mathrm{JJJK}\hat{\mathbf{J}}^{12}\hat{\mathbf{J}}_z^2 + R_\mathrm{J}\hat{\mathbf{J}}^{14} + \{r_\mathrm{K}\hat{\mathbf{J}}_z^{12} + r_\mathrm{KKKJ}\hat{\mathbf{J}}^2\hat{\mathbf{J}}_z^{10} + r_\mathrm{KKJ}\hat{\mathbf{J}}^4\hat{\mathbf{J}}_z^8 + r_\mathrm{JK}\hat{\mathbf{J}}^6\hat{\mathbf{J}}_z^6$$
$$+ r_\mathrm{JJK}\hat{\mathbf{J}}^8\hat{\mathbf{J}}_z^4 + r_\mathrm{JJJK}\hat{\mathbf{J}}^{10}\hat{\mathbf{J}}_z^2 + r_\mathrm{J}\hat{\mathbf{J}}^{12}, \hat{\mathbf{J}}_{xy}^2\}.$$

In the $\hat{\mathbf{H}}_2, \hat{\mathbf{H}}_4, ...., \hat{\mathbf{H}}_{14}$ terms, (a) $\hat{\mathbf{J}}_x$, $\hat{\mathbf{J}}_y$, and $\hat{\mathbf{J}}_z$ are the $x$, $y$, and $z$ components of the total angular momentum operator, respectively, (b) $\hat{\mathbf{J}}^2 = \hat{\mathbf{J}}_x^2 + \hat{\mathbf{J}}_y^2 + \hat{\mathbf{J}}_z^2$, (c) $\hat{\mathbf{J}}_{xy}^2 = \hat{\mathbf{J}}_x^2 - \hat{\mathbf{J}}_y^2$, (d) $\{,\}$ denotes the anticommutator, and (e) the symbols in front of the products of $\hat{\mathbf{J}}_x$, $\hat{\mathbf{J}}_y$, $\hat{\mathbf{J}}_z$, and $\hat{\mathbf{J}}^2$ are the rotational parameters of $\hat{\mathbf{H}}$. The matrix representation of $\hat{\mathbf{H}}$, denoted as $\mathbf{H}$, is block diagonal in the quantum number $J$, and it can be formed by using Wang-type rotational basis functions[13]. The eigenvalues of $\mathbf{H}$ correspond to the energies of the rotational states within the $(000)$ vibrational band, while the rotational assignments can be derived from the symmetry of the Wang functions (for technical details, see Ref. 14).

The optimal rotational parameters can be determined by minimizing the following (non-quadratic) objective function:

$$\Omega(\mathbf{p}) = \sum_{i=1}^{N_\mathrm{T}} \frac{1}{u^2(\sigma_i)} \left[\sigma_i - E_{\mathrm{up}(i)}^{\mathrm{EH}}(\mathbf{p}) + E_{\mathrm{low}(i)}^{\mathrm{EH}}(\mathbf{p})\right]^2 \tag{7}$$

where (a) $\mathbf{p} = \{p_1, p_2, ..., p_{N_\mathrm{P}}\}^\mathrm{T}$ is the vector of the $N_\mathrm{P}$ rotational parameters, (b) $\sigma_i$ is the wavenumber of the $i$th pure rotational line with its $u(\sigma_i)$ uncertainty, (c) $N_\mathrm{T}$ is the number of rotational transitions, (d) $E_j^{\mathrm{EH}}$ is the effective-Hamiltonian-based energy value of the $j$th state involved in rotational lines, and (e) up$(i)$ and low$(i)$ are the indices of the upper and lower energy levels of the $i$th transition, respectively.

A minimum of $\Omega(\mathbf{p})$, $\tilde{\mathbf{p}}$, can be localized, e.g., with the Levenberg–Marquart method[15,16], and the uncertainties of the $\tilde{p}_k$ values can be calculated as[17]

$$u(\tilde{p}_k) = \sqrt{v_{kk}}. \tag{8}$$

In this equation, $v_{kk}$ is a diagonal entry of the $\mathbf{V} = \{v_{kl}\}$ variance-covariance matrix[17], which can be estimated as

$$\mathbf{V} = s_\mathrm{r}^2 \left(\mathbf{D}^\mathrm{T}\mathbf{R}^\mathrm{T}\mathbf{W}\mathbf{R}\mathbf{D}\right)^{-1}, \tag{9}$$

where (a) $s_\mathrm{r}^2 = \mathit{\Omega}(\tilde{\mathbf{p}})/(N_\mathrm{T}-N_\mathrm{P})$ is the residual variance[17], whose square root (residual standard error, $s_\mathrm{r}$) is the goodness factor of the fit, (b) $\mathbf{R} = \{r_{ij}\}$ is the Ritz matrix[18] with

$$r_{ij} = \begin{cases} 1, \text{ if the } j\text{th energy level is the upper state of the } i\text{th line,} \\ -1, \text{ if the } j\text{th energy level is the lower state of the } i\text{th line,} \\ 0, \text{ otherwise,} \end{cases} \tag{10}$$

(c) $\mathbf{D} = \{d_{jk}\}$ is the derivative tensor (Jacobian matrix) with $d_{jk} = \left(\partial E_j^\mathrm{EH}/\partial p_k\right)_{\mathbf{p}=\tilde{\mathbf{p}}}$, and (d) $\mathbf{W}$ is the diagonal matrix of the $1/u^2(\sigma_i)$ weights. Employing the $\mathbf{V}$ matrix, the $u(E_j^\mathrm{EH})$ uncertainties can be approximated as

$$u(E_j^\mathrm{EH}) = [\![\mathbf{DVD}^\mathrm{T}]\!]_{jj}, \tag{11}$$

where $[\![\ ]\!]_{jk}$ returns the $(j,k)$ entry of the matrix in its argument. [Unfortunately, this formula gives too optimistic uncertainties for polynomial models, like Supplementary Equation 6.]

Based on Supplementary Equations 6–10, an in-house effective Hamiltonian code was written to fit pure rotational transitions of $H_2{}^{16}O$ within the ground vibrational state and estimate the lowest *ortho* energy of $H_2{}^{16}O$. As mentioned in the 'Lowest *ortho* energy value' section of the paper, we constructed 111 artificial transitions (from the NICE-OHMS lines and the transitions of Ref. 19) sharing the same upper states and calculated the underlying $(000)$ energy differences with uncertainties. The training set was built upon these artificial lines and pure rotational transitions of Refs. 20, 21, and 22, but excluding those which involve states with $J > 8$ or one of the $(000)1_{1,1}$, $(000)2_{0,2}$, $(000)2_{1,1}$, and $(000)2_{2,0}$ energy levels. The energies of the last four states (external dataset) serve as external validation for our Hamiltonian model. Note that *ortho* states are not placed into the external dataset, as only their relative energies can be derived from the Ritz principle (see Fig. 1 of this paper).

During the fitting procedure, we relied on the following requirements: (a) the residual standard error [see Supplementary Equation 9] of the fit should be as small as possible, (b) the interpolation error has to be minuscule for the energies of the external dataset, (c) $|\mathit{\Delta}_i| \le 2u(\sigma_i)$ should be met for all the lines of the training set, where $\mathit{\Delta}_i = \sigma_i - E_{\mathrm{up}(i)}^\mathrm{EH}(\tilde{\mathbf{p}}) + E_{\mathrm{low}(i)}^\mathrm{EH}(\tilde{\mathbf{p}})$ is the fitting residual, (d) the estimated uncertainties of the fitted parameters must be smaller than 25 % of the parameter values themselves, and (e) the smallest number of rotational parameters must be used in the model. Until these criteria are not satisfied, we added further terms for $\hat{\mathbf{H}}$ or excluded the existing ones to reach a perfect fit to the data.

This way we obtained a 14th-order Hamiltonian model, fixing some (19) parameters ($Q_{\mathrm{JK}}$, $Q_{\mathrm{JJK}}$, $Q_{\mathrm{JJJK}}$, $Q_{\mathrm{J}}$, $q_{\mathrm{JK}}$, $q_{\mathrm{JJK}}$, $q_{\mathrm{J}}$, $R_{\mathrm{KKJ}}$, $R_{\mathrm{KJ}}$, $R_{\mathrm{JK}}$, $R_{\mathrm{JJK}}$, $R_{\mathrm{JJJK}}$, $R_{\mathrm{J}}$, $r_{\mathrm{KKKJ}}$, $r_{\mathrm{KKJ}}$, $r_{\mathrm{JK}}$, $r_{\mathrm{JJK}}$, $r_{\mathrm{JJJK}}$, and $r_{\mathrm{J}}$) at zero in Supplementary Equations Ta–Tg and optimizing all the other ones (44). The output of the fit is reported in sd5.txt. Applying this newly parametrized rotational Hamiltonian leads to an estimate of 23.794 361 22 $\mathrm{cm}^{-1}$ for the lowest *ortho* energy of $H_2{}^{16}O$. As it is obvious from file sd5.txt, our effective Hamiltonian calculations deviate from the experimental energies of the external dataset [that is, 37.137 125 51(24), 70.090 816 14(22), 95.175 940 95(25), and 136.163 920 85(14) $\mathrm{cm}^{-1}$] by $2.3 \times 10^{-8}$, $1.3 \times 10^{-7}$, $-2.8 \times 10^{-8}$, and $6.6 \times 10^{-8}$ $\mathrm{cm}^{-1}$, respectively, which are significantly smaller than the indicated experimental energy uncertainties. Thus, it is expected that the accuracy of the fitted lowest *ortho* energy estimate is not worse than the largest uncertainty of the external dataset, i.e., $2.5\times10^{-7}$ $\mathrm{cm}^{-1}$. This uncertainty approximation is more conservative than the value deduced from Supplementary Equation 11, $4.6 \times 10^{-8}$ $\mathrm{cm}^{-1}$.

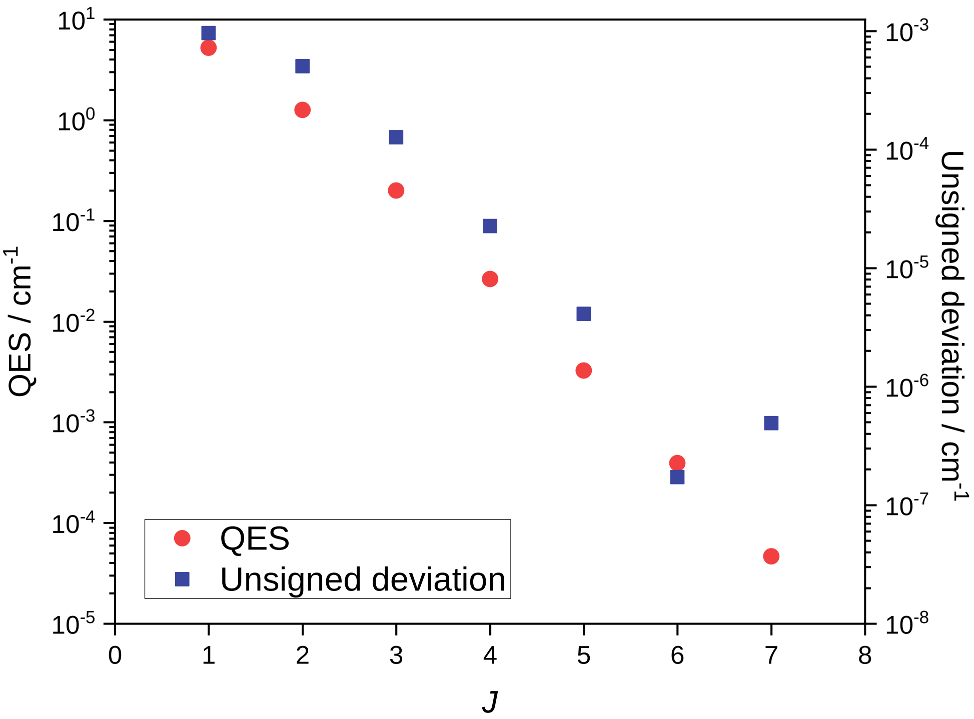


**Supplementary Figure 2**: **Comparison of quasi-experimental and first-principles splitting values for the** $(000)J_{J,0/1}$ **state pairs.** The quasi-experimental splittings (QES) were calculated from our extremely precise relative energies and our effective-Hamiltonian-based lowest *ortho* energy value [i.e., 23.794 361 22(25) $\mathrm{cm}^{-1}$]. The QES values are accurate to some $10^{-7}$ $\mathrm{cm}^{-1}$ (the individual QES uncertainties are irrelevant here: if the need arises, they can be calculated from the law of error propagations and the reported uncertainties of the corresponding energy values). The red dots denote the quasi-experimental splittings as a function of $J$, while the blue squares correspond to the unsigned deviations of the theoretical splittings[23] from their quasi-experimental counterparts. The strange behavior at $J = 6$ and $J = 7$ in the trend of unsigned deviations can be attributed to the fact that the first-principles energy values of Ref. 23 are rounded to six decimal digits.

**Supplementary Note 3 : Uncertainty analysis of close first-principles *ortho–para* splittings**

For the network-theoretical estimation of the lowest *ortho* energy, we need to ascertain that the first-principles computations (providing rovibrational energies typically accurate to a few 0.1 $\mathrm{cm}^{-1}$) can produce high-precision $(v_1v_2v_3)J_{J,0/1}$ splittings at large $J$ values. Thus, we made a comparison of first-principles[23] $(000)J_{J,0/1}$ splittings with our quasi-experimental determinations (obtained from our extremely precise relative energies and our effective-Hamiltonian-based lowest *ortho* energy value [see Supplementary Note 2]).

The splitting values and the deviations between the first-principles and quasi-experimental dataset are depicted in Supplementary Figure 2. It is clear from this figure that, upon the increase of $J$, both the splittings and the deviations between the first-principles and quasi-experimental values are getting smaller, even down to the $10^{-6}$ $\mathrm{cm}^{-1}$ limit represented by the number of decimal digits reported in Ref. 23 for the rovibrational energies. This means that the theoretical computations become ever more accurate with the decrease of the splitting values, allowing the accurate estimation of the $(000)J_{J,0/1}$ splittings at sufficiently large $J$ values.

A further observation can be made by matching the $(101)7_{7,0/1}$ quasi-experimental splitting [$8.9875(67) \times 10^{-4}$ $\mathrm{cm}^{-1}$] with its theoretical[23] counterpart [$8.97 \times 10^{-4}$ $\mathrm{cm}^{-1}$]: the unsigned deviation between the two estimates is only $1.75 \times 10^{-6}$ $\mathrm{cm}^{-1}$. As a result, we are confident that the tiny $(v_1v_2v_3)J_{J,0/1}$ splittings can be reliably determined (at higher $J$ values) from first-principles computations even for highly excited vibrational bands (provided that the splitting values become indeed very small).

**Supplementary Note 4 : GENIUSH computations for virtual lines**

The wavenumbers of the $(040)7_{7,0} \leftarrow (040)7_{7,1}$ and $(040)8_{8,0} \leftarrow (040)8_{8,1}$ virtual lines included in Fig. 5 of the paper were estimated by using the GENIUSH[24,25] variational nuclear-motion code. During our computations, several different basis sets and potential energy surfaces (PES) were combined to obtain reliable wavenumber predictions with definite uncertainties.

The rovibrational Hamiltonian can be expressed with arbitrary internal coordinates in GENIUSH. The matrix representation of the vibrational Hamiltonian is constructed in the discrete variable representation (DVR)[26,27]. The vibrational basis is expressed as the direct product of DVR basis functions[28,29] corresponding to each internal degree of freedom. One can choose the type and the number of DVR basis functions and the range of the coordinate grid. As to rotational motions, the full rovibrational Hamiltonian is block diagonal in the $J$ total angular ro-

tational quantum number. The rotational part of the wavefunction is expressed by $2J + 1$ Wang functions[13] constructed as a linear combination of the symmetric-top rigid-rotor eigenfunctions. This rotational basis forms a complete basis set for a particular $J$. The total rovibrational wavefunction is obtained as the direct product of the vibrational and the rotational parts.

During the computation of the rovibrational energies of $H_2{}^{16}O$, valence coordinates were used in the following ranges: $[0.5, 2.0]$ Å for the bond lengths and $(0, \pi)$ for the bond angle. Four computations were performed with each PES and using 20 and 30 Laguerre PO-DVR functions[29] for both bond length coordinates together with 50 and 80 Legendre DVR functions[28] for the bond angle coordinate. The nuclear masses employed in the computations are the following: $m(\mathrm{O}) = 15.990526$ u and $m(\mathrm{H}) = 1.00727647$ u. The literature PESs adopted for the nuclear-motion computations are CVRQD[30,31], BT2[23,32], PoKaZaTeL[33], PES15k[34], Oka[35,36], Bubukina[36,37], and PS[38].

The $(040)7_{7,0}$, $(040)7_{7,1}$, $(040)8_{8,0}$, and $(040)8_{8,1}$ energies computed with different potentials differ by about 1 $\mathrm{cm}^{-1}$. The convergence of these energies obtained with different basis sets varies from some $10^{-5}$ to a few $10^{-3}$ $\mathrm{cm}^{-1}$.

As observed in Supplementary Note 3, the theoretically computed energies (although their absolute accuracy is insufficient for precision-spectroscopy applications) can be utilized to obtain highly (a few times $10^{-7}$ $\mathrm{cm}^{-1}$) accurate wavenumbers for the virtual lines due to the following factors: (a) errors coming from the imperfect treatment of the vibrations (incomplete basis set, differences in the PESs) cancel each other out when subtracting the energies of the rovibrational states with the same vibrational parent, (b) there is no error in the treatment of the rotation owning to the use of the complete basis set of Wang functions, and (c) the same $J$ values of the investigated pairs also help in the error cancellation. This is the reason why the wavenumbers of the virtual transitions are less sensitive to the choice of the PES and the vibrational basis set than the rovibrational energies themselves.

The predicted wavenumbers of the $(040)7_{7,0} \leftarrow (040)7_{7,1}$ and $(040)8_{8,0} \leftarrow (040)8_{8,1}$ virtual lines obtained with the different basis sets and PESs are presented in file sd7.txt. The final wavenumber values are calculated as the average of the results related to the different basis sets and PES, while the wavenumber uncertainties are approximated as two times the standard deviations of the estimates provided by the various computations. This statistical model gives rise to $4.26(18) \times 10^{-6}$ $\mathrm{cm}^{-1}$ and $2.99(17) \times 10^{-6}$ $\mathrm{cm}^{-1}$ for the wavenumbers of the $(040)7_{7,0} \leftarrow (040)7_{7,1}$ and $(040)8_{8,0} \leftarrow (040)8_{8,1}$, transitions respectively.

**Supplementary Note 5 : Comparison of the various lowest *ortho*-energy estimates**

To make a detailed comparison of the present and previous estimates of the lowest *ortho* energy of $H_2{}^{16}O$, the literature was searched for such estimates and effective Hamiltonian parameters implicitly defining the lowest *ortho* energy. These predictions, as well as our effective-Hamiltonian and path-based values, are collated in file sd8.txt. Our effective-Hamiltonian-based estimate is suggested as a new reference value for the lowest *ortho* energy due to its two times higher accuracy; thus, no further analysis is performed for the insignificant differences between the three predictions of this study. In what follows, our energy estimates for $(000)1_{0,1}$, $(000)1_{1,1}$, $(000)2_{0,2}$, $(000)2_{1,1}$, and $(000)2_{2,0}$ (see files sd4.txt, sd5.txt, and sd6.txt) are called benchmark energies, while the last four states are referred to as verifying energy levels.

In cases when the uncertainties of the lowest *ortho*-energy estimates are not available from the data sources, these uncertainties were calculated as

$$u(E^{\mathrm{lit}}\left[(000)1_{0,1}\right]) = \sqrt{\frac{\delta^2\left[(000)1_{1,1}\right] + \delta^2\left[(000)2_{0,2}\right] + \delta^2\left[(000)2_{1,1}\right] + \delta^2\left[(000)2_{2,0}\right]}{4}}, \quad (12)$$

where $\delta[X] = E^{\mathrm{lit}}[X] - E[X]$ is the deviation of the $E^{\mathrm{lit}}[X]$ literature energy value from the benchmark energy of the verifying state $X$. This formula provides a feasible way to compare the lowest *ortho*-energy values coming from the present and previous studies.

72LuHeCoGo[39] is a source where the submillimeter-wave spectrum of $H_2{}^{16}O$ was interpreted by fitting a 10th-order Watsonian to measured pure rotational transitions within the ground vibrational state. (Since almost the same parametrization is published in 74LuHeKi[40], this latter source is not considered in the present analysis.) Despite the fact that the fitted Hamiltonian of 72LuHeCoGo reproduces well the frequencies of Table I in 72LuHeCoGo, the energy values of 72LuHeCoGo (and also its lowest *ortho* energy) show larger (a few $10^{-4}$ cm$^{-1}$) deviations from the benchmark energies. These deviations may be attributed to the insufficient number of low-$J$ lines included in the fit. Similar observations can be made for 87BaAlAlPo[41].

In 83MeLuHe[42], some further rotational lines were recorded, and (compared to 72LuHeCoGo) a new (extended) set of rotational parameters was determined to calculate the spectrum of $H_2{}^{16}O$ in the range of $0 - 877$ cm$^{-1}$. In Table III of 83MeLuHe, the lower-state energies are also displayed, but without uncertainties. Therefore, these energies are used to represent the lowest *ortho* energy and calculate its uncertainty for 83MeLuHe. Although the lowest *ortho* energy of 83MeLuHe deviates from our benchmark value by less than $1 \times 10^{-6}$ cm$^{-1}$, this

coincidence is accidental: the energies of 83MeLuHe are dissimilar to a greater extent, $\sim 10^{-5}$ $\mathrm{cm}^{-1}$, from the benchmark energies of the verifying states (see file sd8.txt).

In 85Johns[43], around 1200 observed far-infrared transitions and previous accurate microwave lines were involved in a 12th-order Watsonian fit (with a root-mean-square deviation of $\sim 3\times10^{-6}$ $\mathrm{cm}^{-1}$ for the microwave transitions). As found in the case of 83MeLuHe, the lowest *ortho* energy of 85Johns differs to a smaller extent (by some $10^{-6}$ $\mathrm{cm}^{-1}$) from its benchmark value, than the energies of 85Johns related to the verifying energy levels, which are accurate at most to $2 \times 10^{-5}$ $\mathrm{cm}^{-1}$ (see file sd8.txt).

In 91Toth[44], a Fourier-transform spectrum (1066–2582 $\mathrm{cm}^{-1}$) of $H_2{}^{16}O$ and former accurate literature lines were utilized to obtain a set of energy values with an uncertainty of some $10^{-5}$ $\mathrm{cm}^{-1}$ through a Watsonian fit. The $J < 4$ energy values of the $(000)$ vibrational band, with their uncertainties, can be found in Table 2 of 91Toth, allowing their use in the present comparison. It can be observed in file sd8.txt that the energy uncertainties given in 91Toth are underestimated by a factor of $\sim 2$ for the verifying states.

The 95MaOdIwTs[45] experiments were performed in the region of 0.5–5 THz, and the Hamiltonian operator of 85Johns was reparametrized there for the actual transitions. As seen for 83MeLuHe and 85Johns, the lowest *ortho* energy of 95MaOdIwTs diverges less (only by $2 \times 10^{-6}$ $\mathrm{cm}^{-1}$) from its benchmark value than the energies of 95MaOdIwTs corresponding to the verifying states (that is, by $\sim 1 \times 10^{-5}$ $\mathrm{cm}^{-1}$).

01LaCoCa[46] contains two different lowest *ortho*-energy estimates. The first one, with a claimed accuracy of $1 \times 10^{-5}$ $\mathrm{cm}^{-1}$ and a deviation of $1 \times 10^{-6}$ $\mathrm{cm}^{-1}$ from its benchmark energy, is of unknown origin. This datum occurs in Table 4 of 01LaCoCa as an 'observed' energy value without any further information. The other determination, which can be obtained by adding $1 \times 10^{-5}$ $\mathrm{cm}^{-1}$ to the unknown prediction (see Table 4 of 01LaCoCa), is the result of a fit with a special bending-rotational effective Hamiltonian. File sd8.txt clearly demonstrates that the energies coming from the effective Hamiltonian analysis of 01LaCoCa reproduce the benchmark values of the verifying states with a difference of a few $10^{-5}$ $\mathrm{cm}^{-1}$.

The JPL website[47] stores an energy list, supplied with a timestamp of 'Oct 31 21:54:56 2005', which is obtained from fitting with an Euler-type Hamiltonian. The parametrization of this fit is similar to that reported in 05PiPeMi[48]. Since the lowest *ortho* energy found in the list on the web page is assigned with an uncertainty of $0.000\ 000$ $\mathrm{cm}^{-1}$, we have replaced this rounded value with the RMSD ($8.26 \times 10^{-6}$ $\mathrm{cm}^{-1}$) calculated via Supplementary Equation 12.

This estimate is in close agreement with both the relatively large deviations ($\sim 4 \times 10^{-6}$ cm$^{-1}$) and the JPL energies of the verifying states from our benchmark data.

Hence, this value for the lowest *ortho* energy of the JPL laboratory is in large disagreement with our benchmark value. Similarly large differences are shown by from our benchmark data.

The authors of 14CoMaPi[49] improved their bending-rotational Hamiltonian[46] to achieve a better fit to hundreds of microwave, THz, and far-infrared lines. In the Supplementary Materials of their paper, they reported energies rounded to five decimal digits, signaling that they trust in only five figures after the decimal points. The lowest *ortho* energy of 14CoMaPi deviates by $\sim 1 \times 10^{-6}$ cm$^{-1}$ from our benchmark prediction, while a slightly higher RMSD ($5.47 \times 10^{-6}$ cm$^{-1}$) can be discovered between their and our energy values obtained for the verifying states.

Based on these statistical examinations, one can definitely claim that the previous lowest *ortho*-energy estimates have uncertainties not better than $10^{-5} - 10^{-4}$ cm$^{-1}$, even if their actual deviations from our benchmark value are often smaller. If these actual deviations reflected the real uncertainties of the associated lowest *ortho*-energy predictions, the benchmark energies of the verifying states would be better reproduced by the underlying Hamiltonian models.

**Supplementary Note 6 : Generation and the use of frequency standards**

Utilizing the energy levels from file sd4.txt, we derived all the possible $(v_1'v_2'v_3')J'_{K_a',K_c'} \leftarrow (v_1''v_2''v_3'')J''_{K_a'',K_c''}$ lines linking these energy levels and satisfying the following selection rules:

$$|J' - J''| \leq 1, \tag{13}$$

$$(-1)^{K_c''} \neq (-1)^{K_c'}, \tag{14}$$

$$(-1)^{v_3''+K_a''+K_c''} = (-1)^{v_3'+K_a'+K_c'}. \tag{15}$$

The wavenumber of a predicted line is obtained by subtracting the upper and lower relative energy values, while its uncertainty is calculated by applying Supplementary Equation 2 for the path which is formed by the disjoint lines of the core-to-state paths (see file sd4.txt) associated with the upper and lower states. These predicted transitions are listed in file sd3.txt. This treatment provides more realistic estimates for the wavenumber uncertainties than using the square root taken from sum-of-squared uncertainties of the upper and lower levels (compare columns 'unc' and 'conv_unc' in file sd3.txt). The predicted lines are also augmented with Einstein-$A$ coefficients and 296 K intensities[23]. Of the 1219 lines predicted with average and maximum uncertainties of $2.94 \times 10^{-7}$ and $6.46 \times 10^{-7}$ cm$^{-1}$, respectively, 867 are not less intense than $10^{-28}$ cm molecule$^{-1}$ (i.e., they can be measured at room temperature).

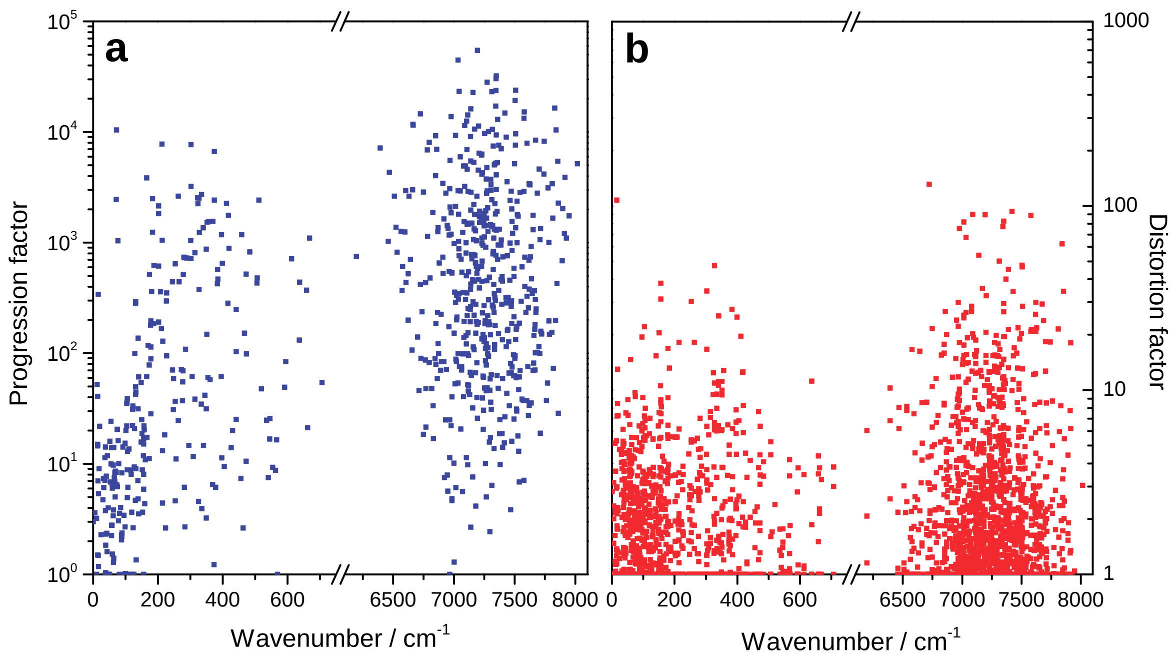


**Supplementary Figure 3**: **Assessment of the literature transitions with respect to lines in file sd3.txt.** (a) Progression factors [reflecting the improvement on the previous line positions; see Supplementary Equation 16] as a function of the wavenumber values. For repeated measurements, only one datum is plotted, namely that one having the smallest progression factor. (b) Distortion factors [representing the relative deviations of the literature line positions from our predictions; see Supplementary Equation 17] versus wavenumbers.

As an interesting utilization, the rovibrational transitions of $H_2{}^{16}O$ were collated from the literature[39,42,43,45,49–116] and matched with the extremely accurate frequency standards of file sd3.txt (except for Refs. 19, 20, 21, and 22, which were ignored during this analysis due to their inclusion in file sd3.txt). These literature lines are listed in file sd10.txt.

This comparison enables us to evaluate the accuracy of 3856 literature transitions, coming from 75 data-source segments (parts of data sources with similar uncertainties) and involving numerous repeated measurements. Out of these matched lines, only 198 are augmented with individual line-position uncertainties. The $i$th literature transition can be characterized with the following factors:

$$\mathrm{PF}_i = \max\left\{\frac{|\sigma_{K_i}^{\mathrm{pred}} - \sigma_i^{\mathrm{lit}}|}{u(\sigma_{K_i}^{\mathrm{pred}})}, 1\right\}, \tag{16}$$

$$\mathrm{DF}_i = \max\left\{\frac{|\sigma_{K_i}^{\mathrm{pred}} - \sigma_i^{\mathrm{lit}}|}{U(\sigma_{K_i}^{\mathrm{pred}} - \sigma_i^{\mathrm{lit}})}, 1\right\}, \tag{17}$$

where (a) $\mathrm{PF}_i$ and $\mathrm{DF}_i$ are the progression and distortion factors of the $i$th literature line, respectively, (b) $\sigma_i^{\mathrm{lit}}$ is the wavenumber of the $i$th literature line, (c) $\sigma_j^{\mathrm{pred}}$ is the wavenumber of the $j$th predicted transition (taken from file sd3.txt), (d) $K_i$ is the index of a predicted line matched

with the $i$th literature transition, (e) $u(\sigma_j^{\text{pred}})$ is the uncertainty of $\sigma_j^{\text{pred}}$, and (f)

$$U(\sigma_{K_i}^{\text{pred}} - \sigma_i^{\text{lit}}) = \sqrt{u^2(\sigma_{K_i}^{\text{pred}}) + U^2(\sigma_i^{\text{lit}})}. \tag{18}$$

In Supplementary Equation 18, the $U(\sigma_i^{\text{lit}})$ parameter is defined as

$$U(\sigma_i^{\text{lit}}) = \begin{cases} u(\sigma_i^{\text{lit}}), & \text{if it is reported,} \\ \text{MUD}^{[\mathfrak{s}_i]}, & \text{otherwise,} \end{cases} \tag{19}$$

where $u(\sigma_i^{\text{lit}})$ is the reported uncertainty of the $i$th literature line (if exists), and $\text{MUD}^{[\mathfrak{s}_i]}$ is the median of the unsigned $|\sigma_{K_j}^{\text{pred}} - \sigma_j^{\text{lit}}|$ deviations for the corresponding $\mathfrak{s}_i$ segment. While the role of the $\text{PF}_i$ values is to gauge the improvement of the line positions with respect to the previous experimental values, the $\text{DF}_i$ factors represent the relative deviations of the literature wavenumbers from their predicted counterparts.

Supplementary Figure 3 displays the PF and DF values of the matched lines. Supplementary Figure 3a clearly shows that most of the transitions (655 out of 782) with non-identical assignments have an improvement better than a factor of 10 (furthermore, 214 lines have progression factors larger than 1000). There are also many lines (1030 out of 3856) which have distortion factors greater than 2 (see Supplementary Figure 3b), while the number of transitions (166) with DF$>$10 is significant, as well.

The large distortion factors of Supplementary Figure 3b may be attributed to (a) systematic effects (like for 95MaOdIwTs[45] and 09CaPuBuTa[94], where the pressure shift was inadequately considered), (b) misprints (e.g., in the 7th line of 13TrKoViPa[103], where a typographic error of 1 MHz is suspected), (c) weak or blended lines (like in 14ReOuMiWa[106]), or (d) issues due to close *ortho-para* doublets (e.g., the 98th and 99th lines of 80KaKy[62]). The detailed analysis of the outlier lines is beyond the scope of this study.

## Supplementary References


1. Császár, A. G. & Furtenbacher, T. Spectroscopic networks. *J. Mol. Spectrosc.* **266**, 99–103 (2011).

2. Furtenbacher, T., Árendás, P., Mellau, G. & Császár, A. G. Simple molecules as complex systems. *Sci. Rep.* **4**, 4654 (2014).

3. Császár, A. G., Furtenbacher, T. & Árendás, P. Small molecules – Big data. *J. Phys. Chem. A* **120**, 8949–8969 (2016).

4. Tóbiás, R., Furtenbacher, T. & Császár, A. G. Cycle bases to the rescue. *J. Quant. Spectr. Rad. Transfer* **203**, 557–564 (2017).

5. Newman, M. E. J. *Networks* (Oxford University Press, Oxford, 2000).

6. Mulliken, R. S. Report on notation for the spectra of polyatomic molecules. *J. Chem. Phys.* **23**, 1997–2011 (1955).

7. Kroto, H. W. *Molecular rotation spectra* (Dover, New York, 1992).

8. Tennyson, J. *et al.* IUPAC critical evaluation of the rotational-vibrational spectra of water vapor. Part III. Energy levels and transition wavenumbers for $H_2{}^{16}O$. *J. Quant. Spectr. Rad. Transfer* **117**, 29–80 (2013).

9. Diestel, R. *Graph Theory* (Springer, Berlin, 2005).

10. Matsushima, F., Tomatsu, N., Nagai, T., Moriwaki, Y. & Takagi, K. Frequency measurement of pure rotational transitions in the $v_2 = 1$ state of $H_2O$. *J. Mol. Spectrosc.* **235**, 190–195 (2006).

11. Watson, J. K. Determination of centrifugal distortion coefficients of asymmetric-top molecules. III. Sextic coefficients. *J. Chem. Phys.* **48**, 4517–4524 (1968).

12. Watson, J. K. Aspects of quartic and sextic centrifugal effects on rotational energy levels. In Durig, J. (ed.) *Vibrational Spectra and Structure*, vol. 6 (Elsevier Scientific Publishing, Amsterdam, 1977).

13. Zare, R. N. *Angular momentum: understanding spatial aspects in chemistry and physics* (Wiley-Interscience, New York, 1988).

14. Kwan, Y. Y. The interacting states of an asymmetric top molecule $XY_2$ of the group $C_{2v}$: Application to five interacting states (101), (021), (120), (200), and (002) of $H_2{}^{16}O$. *J. Mol. Spectrosc.* **71**, 260–280 (1978).

15. Levenberg, K. A method for the solution of certain non-linear problems in least squares. *Q. Appl. Mat.* **2**, 164–168 (1944).

16. Marquardt, D. W. An algorithm for least-squares estimation of non-linear parameters. *J. Soc. Ind. Appl. Math.* **11**, 431–441 (1963).

17. Tasi, G. & Barna, D. Analytical and numerical computation of error propagation of model parameters. *J. Math. Chem.* **49**, 1322–1329 (2011).

18. Árendás, P., Furtenbacher, T. & Császár, A. G. On spectra of spectra. *J. Math. Chem.* **54**, 806–822 (2016).

19. Kassi, S., Stoltmann, T., Casado, M., Daëron, M. & Campargue, A. Lamb dip CRDS of highly saturated transitions of water near 1.4 $\mu$m. *J. Chem. Phys.* **148**, 054201 (2018).

20. Kukolich, S. G. Measurement of the molecular $g$ values in $H_2O$ and $D_2O$ and hyperfine structure in $H_2O$. *J. Chem. Phys.* **50**, 3751–3755 (1969).

21. Golubiatnikov, G. Y., Markov, V. N., Guarnieri, A. & Knochel, R. Hyperfine structure of $H_2{}^{16}O$ and $H_2{}^{18}O$ measured by Lamb-dip technique in the 180–560 GHz frequency range. *J. Mol. Spectrosc.* **240**, 191–194 (2006).

22. Cazzoli, G., Puzzarini, C., Harding, M. E. & Gauss, J. The hyperfine structure in the rotational spectrum of water: Lamb-dip technique and quantum-chemical calculations. *Chem. Phys. Lett.* **473**, 21–25 (2009).

23. Barber, R. J., Tennyson, J., Harris, G. J. & Tolchenov, R. N. A high-accuracy computed water line list. *Mon. Not. R. Astr. Soc.* **368**, 1087–1094 (2006).

24. Mátyus, E., Czakó, G. & Császár, A. G. Toward black-box-type full- and reduced-dimensional variational (ro)vibrational computations. *J. Chem. Phys.* **130**, 134112 (2009).

25. Fábri, C., Mátyus, E. & Császár, A. G. Rotating full- and reduced-dimensional quantum chemical models of molecules. *J. Chem. Phys.* **134**, 074105 (2011).

26. Harris, D. O., Engerholm, G. G. & Gwinn, W. D. Calculation of matrix elements for one-dimensional quantum-mechanical problems and the application to anharmonic oscillators. *J. Chem. Phys.* **43**, 1515–1517 (1965).

27. Light, J. C. & Carrington, T. Discrete variable representations and their utilization. *Adv. Chem. Phys.* **114**, 263–310 (2000).

28. Szalay, V. Discrete variable representations of differential operators. *J. Chem. Phys.* **99**, 1978–1984 (1993).

29. Szidarovszky, T., Császár, A. G. & Czakó, G. On the efficiency of treating singularities in triatomic variational vibrational computations. The vibrational states of $H_3^+$ up to dissociation. *Phys. Chem. Chem. Phys.* **12**, 8373–8386 (2010).

30. Polyansky, O. L. *et al.* High-accuracy ab initio rotation-vibration transitions for water. *Science* **299**, 539–542 (2003).

31. Barletta, P. *et al.* CVRQD ab initio ground-state adiabatic potential energy surfaces for the water molecule. *J. Chem. Phys.* **125**, 204307 (2006).

32. Shirin, S. V., Polyansky, O. L., Zobov, N. F., Barletta, P. & Tennyson, J. Spectroscopically determined potential energy surface of $H_2{}^{16}O$ up to 25 000 $cm^{-1}$. *J. Chem. Phys.* **118**, 2124–2129 (2003).

33. Polyansky, O. L. *et al.* ExoMol molecular line lists XXX: a complete high-accuracy line list for water. *Mon. Not. R. Astr. Soc.* **480**, 2597–2608 (2018).

34. Mizus, I. I. *et al.* High-accuracy water potential energy surface for the calculation of infrared spectra. *Phil. Trans. R. Soc. A* **376**, 20170149 (2018).

35. Polyansky, O. L. *et al.* Calculation of rotation–vibration energy levels of the water molecule with near-experimental accuracy based on an ab initio potential energy surface. *J. Phys. Chem. A* **117**, 9633–9643 (2013).

36. Tennyson, J. (2019). Private communication (PES subroutine from Prof. Tennyson).

37. Bubukina, I. I., Zobov, N. F., Polyansky, O. L., Shirin, S. V. & Yurchenko, S. N. Optimized semiempirical potential energy surface for $H_2{}^{16}O$ up to 26000 $cm^{-1}$. *Optika i Spektroskopiya* **110**, 160–166 (2011).

38. Partridge, H. & Schwenke, D. W. The determination of an accurate isotope dependent potential energy surface for water from extensive ab initio calculations and experimental data. *J. Chem. Phys.* **106**, 4618–4639 (1997).

39. Lucia, F. C. D., Helminger, P., Cook, R. L. & Gordy, W. Submillimeter microwave spectrum of $H_2{}^{16}O$. *Phys. Rev. A* **5**, 487–490 (1972).

40. De Lucia, F. C., Helminger, P. & Kirchhoff, W. H. Microwave spectra of molecules of astrophysical interest v. water vapor. *J. Phys. Chem. Ref. Data* **3**, 211–219 (1974).

41. Baskakov, O. I., Alekseev, V. A., Alekseev, E. A. & Polevoi, B. I. New submillimeter rotational lines of water and its isotopes. *Optika i Spektroskopiya* **63**, 1016–1018 (1987).

42. Messer, J. K., Lucia, F. C. D. & Helminger, P. The pure rotational spectrum of water vapor – a millimeter, submillimeter, and far infrared analysis. *Int. J. Infrared Milli.* **4**, 505–539 (1983).

43. Johns, J. W. C. High-resolution far-infrared ($20-350$ cm$^{-1}$) spectra of several isotopic species of $H_2O$. *J. Opt. Soc. Am. B* **2**, 1340–1354 (1985).

44. Toth, R. A. $v_2$ Band of $H_2{}^{16}O$: line strengths and transition frequencies. *J. Opt. Soc. Am. B* **8**, 2236–2255 (1991).

45. Matsushima, F., Odashima, H., Iwasaki, T., Tsunekawa, S. & Takagi, K. Frequency measure of pure rotational transitions of $H_2O$ from 0.6 to 5 THz. *J. Mol. Spectrosc.* **352**, 371–378 (1995).

46. Lanquetin, R., Coudert, L. H. & Camy-Peyret, C. High-lying rotational levels of water: An analysis of the energy levels of the five first vibrational states. *J. Mol. Spectrosc.* **206**, 54–67 (2001).

47. JPL website. `https://spec.jpl.nasa.gov//ftp//pub/catalog/archive/c018005.egy`. Accessed: 2020-02-26.

48. Pickett, H., Pearson, J. & Miller, C. Use of euler series to fit spectra with application to water. *J. Mol. Spectrosc.* **233**, 174–179 (2005).

49. Coudert, L. H., Martin-Drumel, M.-A. & Pirali, O. Analysis of the high-resolution water spectrum up to the Second Triad and to $J = 30$. *J. Mol. Spectrosc.* **303**, 36–41 (2014).

50. Golden, S., Wentink, T., Hillger, R. & Strandberg, M. W. P. Stark spectrum of $H_2{}^{16}O$. *Phys. Rev.* **73**, 92–93 (1948).

51. Jen, C. K. Rotational magnetic moments in polyatomic molecules. *Phys. Rev.* **81**, 197–203 (1951).

52. Posener, D. W. & Strandberg, M. W. P. Centrifugal distortion effect in asymmetric top molecules III. $H_2O$, $D_2O$, and HDO. *Phys. Rev.* **95**, 374–384 (1954).

53. King, W. C. & Gordy, W. One-to-two millimeter wave spectroscopy. IV. Experimental methods and results for OCS, $CH_3F$, and $H_2O$. *Phys. Rev.* **93**, 407–412 (1954).

54. Hall, R. T. & Dowling, J. M. Pure rotational spectrum of water vapor. *J. Chem. Phys.* **47**, 2454–2461 (1967).

55. Stephenson, D. & Strauch, R. Water vapor spectrum near 600 GHz. *J. Mol. Spectrosc.* **35**, 494–495 (1970).

56. Huiszoon, C. A high resolution spectrometer for the shorter millimeter wavelength region. *Rev. Scient. Instrum.* **42**, 477–481 (1971).

57. Steenbeckeliers, G. & Bellet, J. Spectre micro-onde de molecules $H_2{}^{16}O$, $H_2{}^{17}O$ et $H_2{}^{18}O$. *C. R. Acad. Sc. Paris* **273**, 471–474 (1971).

58. Flaud, J.-M., Camy-Peyret, C. & Valentin, A. Spectre infrarouge a haute résolution des bandes $\nu_1 + \nu_2$ et $\nu_2 + \nu_3$ de $H_2{}^{16}O$. *J. Phys.* **33**, 741–747 (1972).

59. Toth, R. A. & Margolis, J. S. Line positions of $H_2O$ in the 1.33 to 1.45 micron region. *J. Mol. Spectrosc.* **55**, 229–251 (1975).

60. Fleming, J. W. & Gibson, M. J. Far-infrared absorption spectra of water vapor $H_2{}^{16}O$ and isotopic modifications. *J. Mol. Spectrosc.* **62**, 326–337 (1976).

61. Kauppinen, J., Kakkainen, T. & Kyro, E. High-resolution spectrum of water vapour between 30 and 720 $cm^{-1}$. *J. Mol. Spectrosc.* **71**, 15–45 (1978).

62. Kauppinen, J. & Kyro, E. High resolution pure rotational spectrum of water vapor enriched by $H_2{}^{17}O$ and $H_2{}^{18}O$. *J. Mol. Spectrosc.* **84**, 405–423 (1980).

63. Partridge, R. H. Far-infrared absorption spectra of $H_2{}^{16}O$, $H_2{}^{17}O$, and $H_2{}^{18}O$. *J. Mol. Spectrosc.* **56**, 429–437 (1978).

64. Kauppinen, J., Jomena, K. & Horneman, V.-M. New wavenumber calibration tables for $H_2O$, $CO_2$ and OCS lines between 400 $cm^{-1}$ and 900 $cm^{-1}$. *Appl. Opt.* **21**, 3332–3336 (1982).

65. Burenin, A. V., Fevralskikh, T. M., Karyakin, E. N., Polyansky, O. L. & Shapin, S. M. Effective Pade Hamiltonian operator and its application for treatment of $H_2{}^{16}O$ rotational spectrum in the ground state. *J. Mol. Spectrosc.* **100**, 182–192 (1983).

66. Helminger, P., Messer, J. K. & De Lucia, F. C. Continuously tunable coherent spectroscopy for the 0.1-1.0 THz region. *Appl. Phys. Lett.* **42**, 309–310 (1983).

67. Mandin, J.-Y., Chevillard, J.-P., Camy-Peyret, C. & Flaud, J.-M. Line intensities in the $\nu_1 + 2\nu_2$, $2\nu_2 + \nu_3$, $2\nu_1$, $\nu_1 + \nu_3$, $2\nu_3$, and $\nu_1 + \nu_2 + \nu_3 - \nu_2$ bands of $H_2{}^{16}O$, between 6300 and 7900 cm$^{-1}$. *J. Mol. Spectrosc.* **118**, 96–102 (1986).

68. Bauer, A., Godon, M., Kheddar, M. & Hartmann, J. Temperature and perturber dependences of water vapor line-broadening. Experiments at 183 GHz; calculations below 1000 GHz. *J. Quant. Spectr. Rad. Transfer* **41**, 49–54 (1989).

69. Toth, R. A. Extensive measurements of $H_2{}^{16}O$ frequencies and strengths: 5750 to 7965 cm$^{-1}$. *Appl. Opt.* **33**, 4852–4867 (1994).

70. Paso, R. & Horneman, V.-M. High-resolution rotational absorption spectra of $H_2{}^{16}O$, $HD^{16}O$, and $D_2{}^{16}O$ between 110 and 500 cm$^{-1}$. *J. Opt. Soc. Am. B* **12**, 1813–1838 (1995).

71. Markov, V. N. & Krupnov, A. F. Measurements of the pressure shift of the (110)–(101) water line at 556 GHz produced by mixtures of gases. *J. Mol. Spectrosc.* **172**, 211–214 (1995).

72. Brown, L. R. & Plymate, C. Experimental line parameters of the Oxygen A band at 760 nm. *J. Mol. Spectrosc.* **100**, 166–179 (1996).

73. Polyansky, O. L. *et al.* High temperature rotational transitions of water in sunspot and laboratory spectra. *J. Mol. Spectrosc.* **186**, 422–447 (1997).

74. Chance, K., Park, K. & Evenson, K. Pressure broadening of far infrared rotational transitions: 88.65 cm$^{-1}$ $H_2O$ and 114.47 cm$^{-1}$ $O_3$. *J. Quant. Spectr. Rad. Transfer* **59**, 687–688 (1998).

75. Bauer, A., Godon, M., Carlier, J. & Gamache, R. Continuum in the windows of the water vapor spectrum. Absorption of $H_2O$-Ar at 239 GHz and linewidth calculations. *J. Quant. Spectr. Rad. Transfer* **59**, 273–285 (1998).

76. Chen, P., Pearson, J. C., Pickett, H. M., Matsuura, S. & Blake, G. A. Submillimeter-wave measurements and analysis of the ground and $v_2 = 1$ states of Water. *Astrophys. J. Suppl. Ser.* **128**, 371–385 (2000).

77. Bykov, A. *et al.* High-order resonances in the water molecule. *J. Mol. Spectrosc.* **205**, 1–8 (2001).

78. Tereszchuk, K. *et al.* Laboratory spectroscopy of hot water near 2 microns and sunspot spectroscopy in the H-band region. *Astrophys. J.* **577**, 496–500 (2002).

79. Zou, Q. & Varanasi, P. Laboratory measurement of the spectroscopic line parameters of water vapor in the 610–2100 and 3000–4050 cm$^{-1}$ regions at lower-tropospheric temperatures. *J. Quant. Spectr. Rad. Transfer* **82**, 45–98 (2003).

80. Coudert, L. H., Pirali, O., Vervloet, M., Lanquetin, R. & Camy-Peyret, C. The eight first vibrational states of the water molecule: measurements and analysis. *J. Mol. Spectrosc.* **228**, 471–498 (2004).

81. Shirin, S. V. *et al.* Analysis of hot $D_2O$ emission using spectroscopically determined potentials. *J. Chem. Phys.* **120**, 206–210 (2004).

82. Macko, P. *et al.* High sensitivity CW-cavity ring down spectroscopy of water in the region of the 1.5 $\mu$m atmospheric window. *J. Mol. Spectrosc.* **227**, 90–108 (2004).

83. Tolchenov, R. N. & Tennyson, J. Water line parameters for weak lines in the range 7400–9600 cm$^{-1}$. *J. Mol. Spectrosc.* **231**, 23–27 (2005).

84. Toth, R. A. Measurements of positions, strengths and self-broadened widths of $H_2O$ from 2900 to 8000 cm$^{-1}$: line strength analysis of the 2$^{\rm nd}$ triad bands. *J. Quant. Spectr. Rad. Transfer* **94**, 51–107 (2005).

85. Horneman, V.-M., Anttila, R., Alanko, S. & Pietila, J. Transferring calibration from $CO_2$ laser lines to far infrared water lines with the aid of the $v_2$ band of OCS and the $v_2$, $v_1-v_2$, and $v_1+v_2$ bands of $^{13}CS_2$: Molecular constants of $^{13}CS_2$. *J. Mol. Spectrosc.* **234**, 238–254 (2005).

86. Coheur, P.-F. *et al.* 3200 K laboratory emission spectrum of water. *J. Chem. Phys.* **122**, 074307 (2005).

87. Golubiatnikov, G. Y. Shifting and broadening parameters of the water vapor 183 GHz line (313–220) by $H_2O$, $O_2$, $N_2$, $CO_2$, $H_2$, He, Ne, Ar, and Kr at room temperature. *J. Mol. Spectrosc.* **230**, 196–198 (2005).

88. Jenouvrier, A. *et al.* Fourier transform measurements of water vapor line parameters in the 4200–6600 cm$^{-1}$ region. *J. Quant. Spectr. Rad. Transfer* **105**, 326–355 (2007).

89. Mikhailenko, S. N., Le, W., Kassi, S. & Campargue, A. Weak water absorption lines around 1.455 and 1.66 $\mu$m by CW-CRDS. *J. Mol. Spectrosc.* **244**, 170–178 (2007).

90. Koshelev, M. A. *et al.* Broadening and shifting of the 321-, 325- and 380 GHz lines of water vapor by pressure of atmospheric gases. *J. Mol. Spectrosc.* **241**, 101–108 (2007).

91. Mikhailenko, S. *et al.* Water vapor absorption line intensities in the 1900–6600 $cm^{-1}$ region. *J. Quant. Spectr. Rad. Transfer* **109**, 2687–2696 (2008).

92. Zobov, N. F. *et al.* Spectrum of hot water in the 4750–13 000 $cm^{-1}$ frequency range. *Mon. Not. R. Astr. Soc.* **387**, 1093–1098 (2008).

93. Liu, A., Naumenko, O., Kassi, S. & Campargue, A. High sensitivity CW-CRDS of $^{18}O$ enriched water near 1.6 mm. *J. Quant. Spectr. Rad. Transfer* **110**, 1781–1800 (2009).

94. Cazzoli, G., Puzzarini, C., Buffa, G. & Tarrini, O. Pressure-broadening of water lines in the THz frequency region: Improvements and confirmations for spectroscopic databases. Part II. *J. Quant. Spectr. Rad. Transfer* **110**, 609–618 (2009).

95. Koshelev, M. Collisional broadening and shifting of the 211–202 transition of $H_2{}^{16}O$, $H_2{}^{17}O$, and $H_2{}^{18}O$ by atmosphere gases. *J. Quant. Spectr. Rad. Transfer* **112**, 550–552 (2011).

96. Mikhailenko, S., Kassi, S., Wang, L. & Campargue, A. The absorption spectrum of water in the 1.25 $\mu$m transparency window (7408–7920 $cm^{-1}$). *J. Mol. Spectrosc.* **269**, 92–103 (2011).

97. Drouin, B. J., Yu, S., Pearson, J. C. & Gupta, H. Terahertz spectroscopy for space applications: 2.5–2.7 THz spectra of HD, $H_2O$ and $NH_3$. *J. Mol. Struct.* **1066**, 2–12 (2011).

98. S. N. Mikhailenko and O. V. Naumenko and A. V. Nikitin and I. A. Vasilenko and A.-W. Liu and K.-F. Song and H.-Y. Ni and S.-M. Hu. Absorption spectrum of deuterated water vapor enriched by $^{18}O$ between 6000 and 9200 $cm^{-1}$. *J. Quant. Spectr. Rad. Transfer* **113**, 653–669 (2012).

99. Down, M. J., Tennyson, J., Orphal, J., Chelin, P. & Ruth, A. A. Analysis of an $^{18}O$ and D enhanced water spectrum and new assignments for $HD^{18}O$ and $D_2{}^{18}O$ in the near-infrared region (6000-–7000 $cm^{-1}$) using newly calculated variational line lists. *J. Mol. Spectrosc.* **282**, 1–8 (2012).

100. Yu, S. *et al.* Measurement and analysis of new terahertz and Far-infrared spectra of high temperature water. *J. Mol. Spectrosc.* **279**, 16–25 (2012).

101. Leshchishina, O., Mikhailenko, S., Mondelain, D., Kassi, S. & Campargue, A. CRDS of water vapor at 0.1 Torr between 6886 and 7406 $cm^{-1}$. *J. Quant. Spectr. Rad. Transfer* **113**, 2155–2166 (2012).

102. Leshchishina, O., Mikhailenko, S. N., Mondelain, D., Kassi, S. & Campargue, A. An improved line list for water vapor in the 1.5 $\mu$m transparency window by highly sensitive CRDS between 5852 and 6607 $cm^{-1}$. *J. Quant. Spectr. Rad. Transfer* **130**, 69–80 (2013).

103. Tretyakov, M., Koshelev, M., Vilkov, I., Parshin, V. & Serov, E. Resonator spectroscopy of the atmosphere in the 350–500 GHz range. *J. Quant. Spectr. Rad. Transfer* **114**, 109–121 (2013).

104. Yu, S., Pearson, J. C. & Drouin, B. J. Terahertz spectroscopy of water in its second triad. *J. Mol. Spectrosc.* **288**, 7–10 (2013).

105. Osipov, K. Y., Kapitanov, V. A., Protasevich, A. E., Pereslavtseva, A. A. & Ponurovsky, Y. Y. Diode laser spectroscopy of $H_2{}^{16}O$ spectra broadened by $N_2$ and He in 1.39 mm region. *J. Quant. Spectr. Rad. Transfer* **142**, 1–8 (2014).

106. Regalia, L. *et al.* Water vapor line parameters from 6450 to 9400 $cm^{-1}$. *J. Quant. Spectr. Rad. Transfer* **136**, 119–136 (2014).

107. Liu, A. W., Naumenko, V., Kassi, S. & Campargue, A. CW-cavity ring down spectroscopy of deuterated water in the 1.58 $\mu$m atmospheric transparency window. *J. Quant. Spectr. Rad. Transfer* **138**, 97–106 (2014).

108. Campargue, A. *et al.* The absorption spectrum of water vapor in the 1.25 $\mu$m atmospheric window (7911–8337 $cm^{-1}$). *J. Quant. Spectr. Rad. Transfer* **157**, 135–152 (2015).

109. Sironneau, V. T. & Hodges, J. T. Line shapes, positions and intensities of water transitions near 1.28 $\mu$m. *J. Quant. Spectr. Rad. Transfer* **152**, 1–15 (2015).

110. Mikhailenko, S. *et al.* CRDS of 17O enriched water between 5850 and 6671 $cm^{-1}$: more than 1000 energy levels of$H_2{}^{17}O$ and $HD^{17}O$ newly determined. *J. Quant. Spectr. Rad. Transfer* **177**, 108–116 (2016).

111. Mondelain, D. *et al.* Comb-assisted cavity ring down spectroscopy of $^{17}O$ enriched water between 7443 and 7921 $cm^{-1}$. *J. Quant. Spectr. Rad. Transfer* **203**, 206–212 (2017).

112. Rutkowski, L. *et al.* Comb-Assisted Cavity Ring Down Spectroscopy of $^{17}O$ enriched water between 6667 and 7443 $cm^{-1}$. *J. Quant. Spectr. Rad. Transfer* **205**, 213–219 (2018).

113. Czinki, E., Furtenbacher, T., Császár, A. G., Eckhardt, A. K. & Mellau, G. C. The 1943 K emission spectrum of $H_2{}^{16}O$ between 6600 and 7050 $cm^{-1}$. *J. Quant. Spectr. Rad. Transfer* **206**, 46–54 (2018).

114. Mikhailenko, S. N., Mondelain, D., Karlovets, E. V., Kassi, S. & Campargue, A. Comb-assisted cavity ring down spectroscopy of $^{17}$O enriched water between 6667 and 7443 cm$^{-1}$. *J. Quant. Spectr. Rad. Transfer* **206**, 163–171 (2018).

115. Schroeder, P. J. *et al.* Speed-dependent Voigt lineshape parameter database from dual frequency comb measurements up to 1305 K. Part I: Pure $H_2O$ absorption, 6801–7188 cm$^{-1}$. *J. Quant. Spectr. Rad. Transfer* **210**, 240–250 (2018).

116. Régalia, L., Thomas, X., Rennesson, T. & Mikhailenko, S. Line parameters of water vapor enriched by $^{18}$O from 6525 to 8011 cm$^{-1}$. *J. Quant. Spectr. Rad. Transfer* **235**, 257–271 (2019).
